# Production, Purification, and Applications of a Potential Theranostic Pair: Cobalt-55 and Cobalt-58m

**DOI:** 10.3390/diagnostics11071235

**Published:** 2021-07-09

**Authors:** Kendall E. Barrett, Hailey A. Houson, Wilson Lin, Suzanne E. Lapi, Jonathan W. Engle

**Affiliations:** 1 Department of Medical Physics, University of Wisconsin, 1111 Highland Avenue, Madison, WI 53711, USA; kbarrett3@wisc.edu (K.E.B.); wlin99@wisc.edu (W.L.); 2 Department of Radiology, University of Alabama at Birmingham, 619 19th Street, Birmingham, AL 35294, USA; hhouson@uabmc.edu (H.A.H.); lapi@uab.edu (S.E.L.); 3 Department of Radiology, University of Wisconsin, 600 Highland Avenue, Madison, WI 53792, USA

**Keywords:** cobalt-55, cobalt-58m, radionuclide production, positron emission tomography, targeted radionuclide therapy

## Abstract

The emerging success of [^68^Ga/^177^Lu]Ga/Lu-DOTATATE as a theranostic pair has spurred interest in other isotopes as potential theranostic combinations. Here, we review cobalt-55 and cobalt-58m as a potential theranostic pair. Radionuclidically pure cobalt-55 and cobalt-58m have been produced on small cyclotrons with high molar activity. In vitro, DOTATOC labeled with cobalt has shown greater affinity for SSTR2 than DOTATOC labeled with gallium and yttrium. Similarly, [^58m^Co]Co-DOTATATE has shown improved cell-killing capabilities as compared to DOTATATE labeled with either indium-111 or lutetium-177. Finally, PET imaging with an isotope such as cobalt-55 allows for image acquisition at much later timepoints than gallium, allowing for an increased degree of biological clearance of non-bound radiotracer. We discuss the accelerator targetry and radiochemistry used to produce cobalt-55,58m, emphasizing the implications of these techniques to downstream radiotracers being developed for imaging and therapy.

## 1. Introduction

Radiopharmaceuticals are clinically useful for the diagnosis and treatment of disease [1,2,3,4,5]. However, quantifying radiopharmaceuticals’ dosimetry is challenging [6]. Matching diagnostic radionuclides with chemically similar therapeutics creates a “theranostic” pair, helping to resolve this problem [7]. The success of theranostic pairs has led to the re-evaluation of several radionuclides as potential radiopharmaceuticals, with the most popular in recent times being gallium-68 (t_1/2_ = 68 min, β^+^ = 89%, E_max_= 1899 keV) and lutetium-177 (t_1/2_ = 6.7 d, E_β-_ = 134 keV) [8]. The availability of both gallium-68 and lutetium-177 and the success of conjugating a myriad of targeting vectors to them have led to their widespread adoption in the nuclear medicine community. However, several shortcomings exist for these two radionuclides; namely, the short half-life of gallium-68 limits the range of its applicability, and the resistance of some cancers to lutetium-177-labelled radiopharmaceuticals has prompted a search for other therapeutic radionuclides [9]. Additionally, although gallium-68 and lutetium-177 have been used as a theranostic pair, slight chemical differences between the two may complicate dosimetric calculations and/or lead to subtle biological incongruities [10]. Using compounds labelled with the same chemical element can obviate this problem and allow the same protocols to be used in forming and purifying chemical complexes [11]. Cobalt-55 (t_1/2_ = 17.53 h, β^+^ = 77%, E_γ_ = 931.1 keV, I_γ_ = 75%) and cobalt-58m (t_1/2_ = 9.10 h, IC= 100%) are one of the most promising of such pairs identified due to their identical chemical properties coupled to similar half-lives and high in vivo stability and compatibility with many chelators which can be coupled with targeting biomolecules [8,12,13,14,15,16,17]. We review the production and application of cobalt-55,58m with emphasis placed on potential translational and clinical applications.

## 2. Irradiation

Both cobalt-55 and cobalt-58m are produced primarily from charged particle irradiation of iron or nickel targets. The availability of deuterons likely increases the purity of achievable cobalt-55 but has less bearing on the production of cobalt-58m except for from a target material cost perspective. Practically speaking, clinical application will always have to carefully consider target isotopic enrichment, but target fabrication and recovery are well developed (and reported) processes. These processes are described in greater detail below, treating the nuclear reactions relevant to each radionuclide separately. It should be stated that all radiation decay information comes from Brookhaven National Lab NuDat database [17].

### 2.1. Cobalt-55

Cobalt-55 production was reported in 1938 from alpha irradiation of manganese [18] and has since been reported from many target materials and incident beams. Practically, two reactions are best suited for producing activities of cobalt-55 with few impurities: ^58^Ni(p,α)^55^Co and ^54^Fe(d,n)^55^Co, which are accessible on low energy cyclotrons [19]. These reactions have been thoroughly and recently reviewed by the International Atomic Energy Agency and significant independent efforts resulting in recommended excitation functions for the reactions discussed below [20].

The ^58^Ni(p,α)^55^Co reaction has been employed by many groups [14,21,22,23,24]. Several radioisotopic and radionuclidic impurities can be formed in this process. Cobalt-57(t_1/2_ = 271.7 d, E_γ_= 1377 keV, I_γ_ = 81.7%) is produced from ^58^Ni(p,2p)^57^Co above 15 MeV [23], and production of cobalt-56 occurs above 25 MeV [25]. Nickel-57 (t_1/2_ = 35.68 h, β^+^ = 44%, E_γ_= 1377.6 keV, I_γ_ = 81.7%) is primarily produced via the ^58^Ni(p,2p)^57^Cu, which decays to nickel-57 with a half-life of 196 ms [21]. This reaction occurs above the threshold of 12 MeV, and is useful for monitoring separation efficiency during process development. However, nickel-57 decays to cobalt-57, which cannot be separated from cobalt-55. Natural nickel targets can also be used, but result in increased impurities that limit downstream clinical work [26].

**Figure 1:**
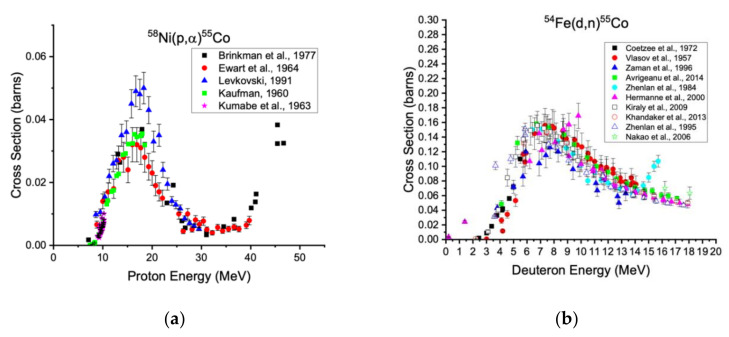
Cross section data for the production of cobalt-55 via the ^58^Ni(p,α)^55^Co (**a**) and ^54^Fe(d,n)^55^Co reactions (**b**) [20].

Cobalt-55 can also be produced by the ^54^Fe(d,n)^55^Co reaction [27]. Theoretically, this method affords radioisotopically pure cobalt-55 compared with the proton-induced route described above, avoiding cobalt-56 and cobalt-57. Practically, purities are limited by the achievable enrichment of the iron-56 target. The excitation function peaks near 7 MeV (Figure 1) [28]. Finally, manganese-52m is also coproduced via the ^54^Fe(d,α)^52m^Mn reaction, which has a threshold energy of 4 MeV [28].

### 2.2. Cobalt-58m: Fe Targets

**Figure 2:**
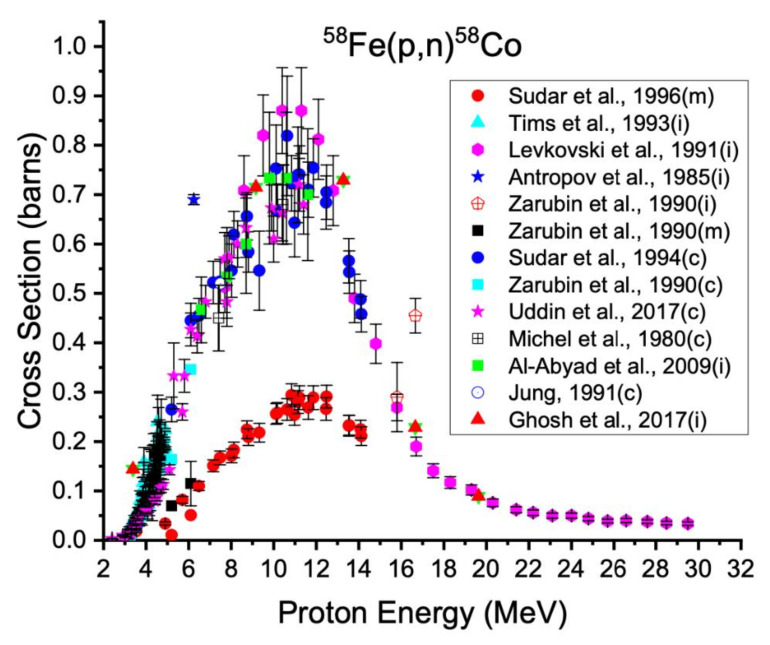
Cross section data for production of cobalt-58m and cobalt-58g from iron-58 [29,30,31,32,33,34,35,36,37,38,39].

The highest-yielding reaction routes for relatively common cyclotrons with dual particle capabilities use proton or deuteron bombardment on iron targets. Cobalt-58m can be produced via the (p,n) reaction on iron-58, which has a natural isotopic abundance of 0.3%. Measured data for the excitation functions ^58^Fe(p,n)^58m^Co and ^58^Fe(p,n)^58g^Co (t_1/2_ = 70.9 d, β^+^ = 14.94%, E_ave β+_ = 475 keV) are shown in Figure 2 below.

In Figure 2, “m” indicates measurements of the direct formation of cobalt-58m, “c” indicates cumulative formation of cobalt-58g either after the complete decay of the isomer or with simultaneous accounting for the contribution from cobalt-58m decay, and “i” indicates measurement of the independent formation of cobalt-58g without contribution from cobalt-58m. The isomers cobalt-58m and cobalt-58g are co-produced in any charged particle irradiation. While the production of cobalt-58g is unavoidable, other radioisotopic impurities can be reduced with careful modulation of proton energies and target material enrichments.

The ^58^Fe(p,2n)^57^Co (t_1/2_ = 271.7 d, E_γ_= 122.1 keV, I_γ_ = 85.6%) and ^58^Fe(p,3n)^56^Co (t_1/2_ = 77.2 d, β^+^ = 19.5%, E_γ_= 846.8 keV, I_γ_ = 99.9%) nuclear reactions have a higher energy threshold than ^58^Fe(p,n)^58^Co, seen in Table 1 below [40].

**Figure 3:**
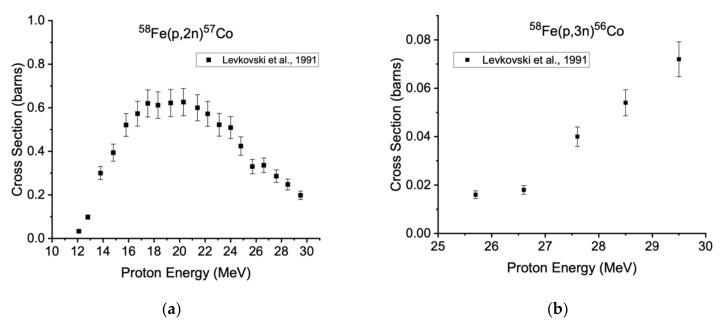
Previously reported excitation functions for (**a**) ^58^Fe(p,2n)^57^Co and (**b**) ^58^Fe(p,3n)^56^Co, measured using isotopically enriched targets [32].

The peak cross section for the production of cobalt-58 is between 10 and 15 MeV. However, the maxima of the ^58^Fe(p,2n)^57^Co and ^58^Fe(p,3n)^56^Co reactions, as shown in Figure 3, occur between 17 and 21 and >30 MeV, respectively [32]. Degrading the proton beam captures the maximum of the ^58^Fe(p,n)^58m^Co excitation function while minimizing the radioisotopic impurity-producing reactions.

**Figure 4:**
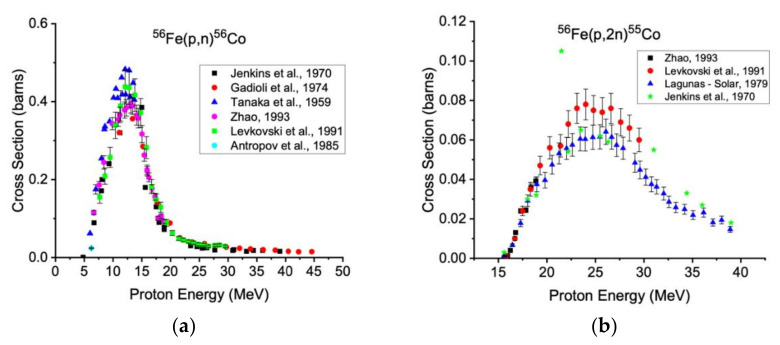
Measured excitation functions for ^56^Fe(p,n)^56^Co (**a**) and ^56^Fe(p,2n)^55^Co (**b**) [32,33,41,42,43,44,45]. These data are in agreement with measured data for production of cobalt-56 up to the threshold of the ^57^Fe(p,2n)^56^Co reaction and for production of cobalt-55 up to the threshold of the ^57^Fe(p,3n)^55^Co reaction using natural Fe targets [35,36,38,39,44,46,47,48,49,50,51,52,53,54,55].

**Figure 5:**
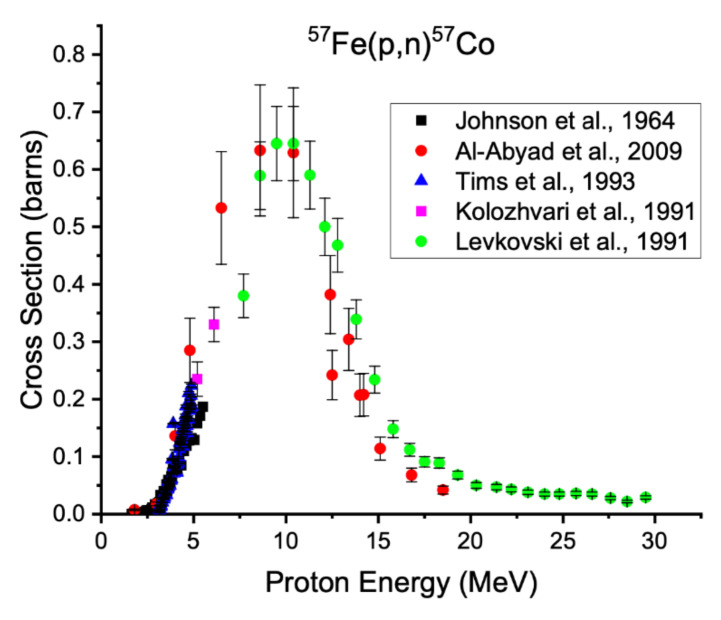
Measured excitation functions for the ^57^Fe(p,n)^57^Co reaction [30,32,38,56,57].

Irradiating isotopically enriched iron-58 avoids the production of other impurities that cannot be separated out in the downstream chemistry. Natural iron is composed of iron-54 (5.8%), iron-56 (91.7%), iron-57 (2.2%), and iron-58 (0.3%). Proton bombardment of iron-54 leads to the production of very short-lived contaminants such as ^54^Fe(p,n)^54^Co (t_1/2_ = 1.46 m) and ^54^Fe(p,2n)^53^Co (t_1/2_ = 0.24 s) that do not diminish the radioisotopic purity of cobalt-58m. However, higher-energy proton reactions on iron-56 and iron-57, as described above for iron-58, may form longer-lived radioisotopic impurities cobalt-56 and cobalt-57. Measured data from the relevant excitation functions are shown below in Figure 4 and Figure 5.

Deuteron bombardments can be used to produce cobalt-58m from enriched iron-57 via the (d,n) reaction, natural iron targets via the (d,n) reaction on iron-57 (2.2% abundance) or (d,2n) reaction on iron-56 (91.7%). Higher stopping power for deuterons reduces the quantity of isotopically enriched target material required, and deuteron-induced reactions’ excitation functions are often higher than similar reactions incited by proton beams. However, the relative thermal power density deposited in the target by the deuteron beam is higher than for protons. Accelerators with deuteron beams are also less common, and where they do exist, they usually have energies too low to utilize the entirety of even (d,n) excitation functions. Deuteron irradiations to produce cobalt-58m proceed on iron isotopes iron-57 and iron-58, which both exist in relatively low abundance, so as with proton irradiations, significant costs are associated with target materials.

**Figure 6:**
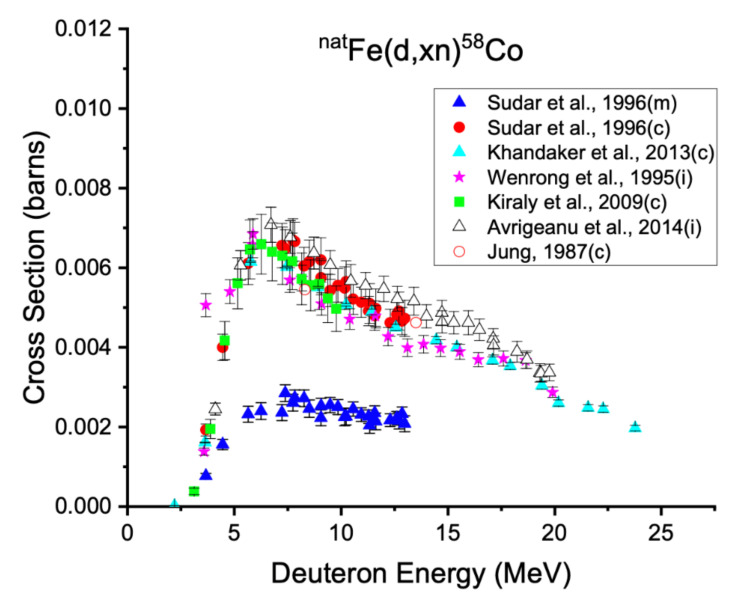
Excitation functions for the production of cobalt-58m,g from deuteron bombardment on natural iron targets. While ^57^Fe(d,n)^58^Co reaction is the dominant one, according to Sudar et al., ^56^Fe(d,γ)^58^Co and ^58^Fe(d,2n)^58^Co data also contribute to the measured excitation functions [29,58,59,60,61,62].

**Figure 7:**
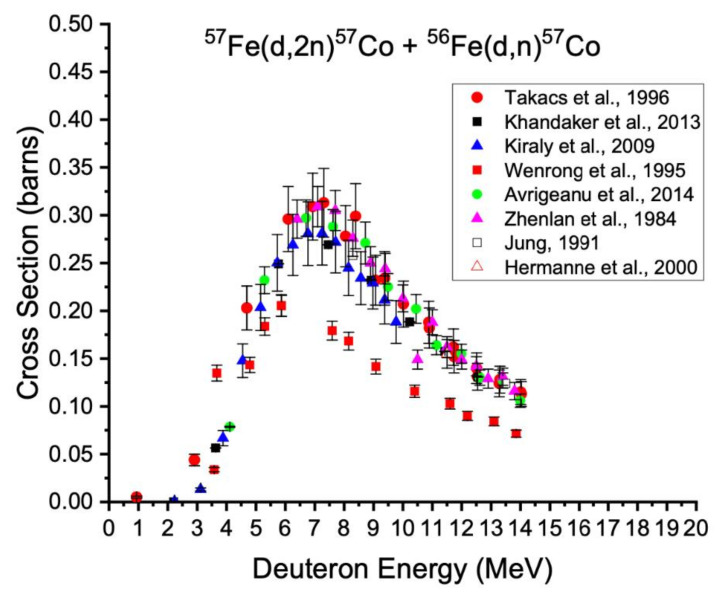
Excitation functions for the production of cobalt-57 from the ^57^Fe(d,2n) and ^56^Fe(d,n) nuclear reactions [58,59,61,62,63,64,65].

As in Figure 2, in Figure 6 “m” indicates measurements of the direct formation of cobalt-58m, “c” indicates cumulative formation of cobalt-58g either after the complete decay of the isomer or with simultaneous accounting for the contribution from cobalt-58m decay, and “i” indicates measurement of the independent formation of cobalt-58g without contribution from cobalt-58m. Figure 6 above plots the excitation functions of ^nat^Fe(d,xn) reactions that form cobalt-58m/g and enriched iron-57 can be used to increase yields and purities. Using lower-energy deuterons reduces the likelihood of the ^57^Fe(d,2n)^57^Co reaction occurring, which has a threshold energy of 3.9 MeV [40]. Overall, an enriched iron-57 target reduces radioisotopic impurities within the cobalt-58m sample. The possible ^57^Co-producing reactions excitation functions are shown in Figure 7. Sudar and Qaim examined the production ratio of the ground and metastable isomers of cobalt-58 from deuteron irradiations of natural iron targets [29] based on the assumptions that deuteron capture and iron-58 target reactions’ contributions are small, but to our knowledge, no measurements have been made of the formation of cobalt-58m/g using separated iron-57.

In Figure 7, an energy of 14.4 MeV is used as a cutoff to remove any data possibly acquired from ^58^Fe(d,3n)^57^Co reactions (threshold energy of 14.4 MeV). However, the threshold energy for ^57^Fe(d,2n)^57^Co is 3.9 MeV, and therefore it is difficult to differentiate between the production via the ^56^Fe(d,n) and ^57^Fe(d,2n) reactions [40].

### 2.3. Cobalt-58m: Ni Targets

**Figure 8:**
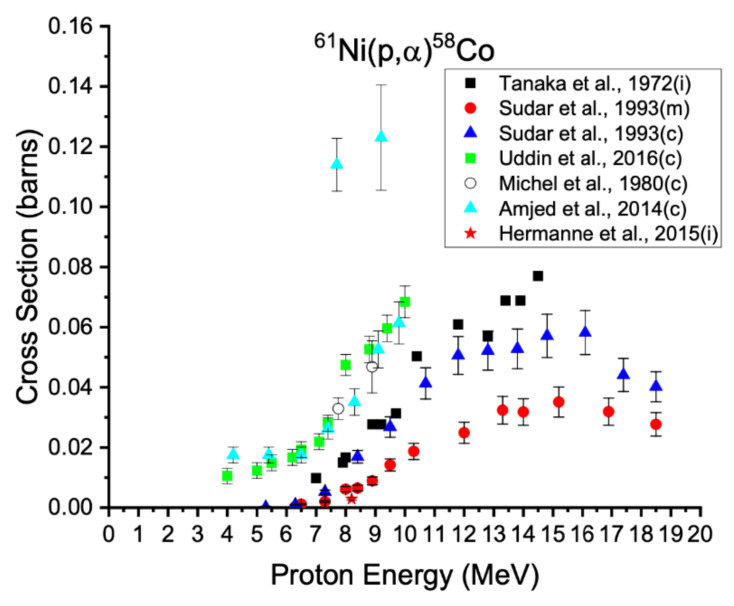
Excitations functions for the production of cobalt-58 on nickel-61 targets [37,66,67,68,69,70]. Data reported by Uddin et al., Michel et al., Amjed et al., and Hermanne et al. are from the irradiation of natural nickel normalized to the abundance of nickel-61, where data beyond the threshold energy for ^61^Ni(p,nα), 10.3 MeV, were not considered.

Nickel targets can also be irradiated with protons to produce cobalt-58m via the ^61^Ni(p,α)^58m^Co nuclear reaction (see Figure 8). Natural nickel has 5 stable isotopes: nickel-58 (68.1%), nickel-60(26.2%), nickel-61 (3.6%), and nickel-64 (0.93%).

**Figure 9:**
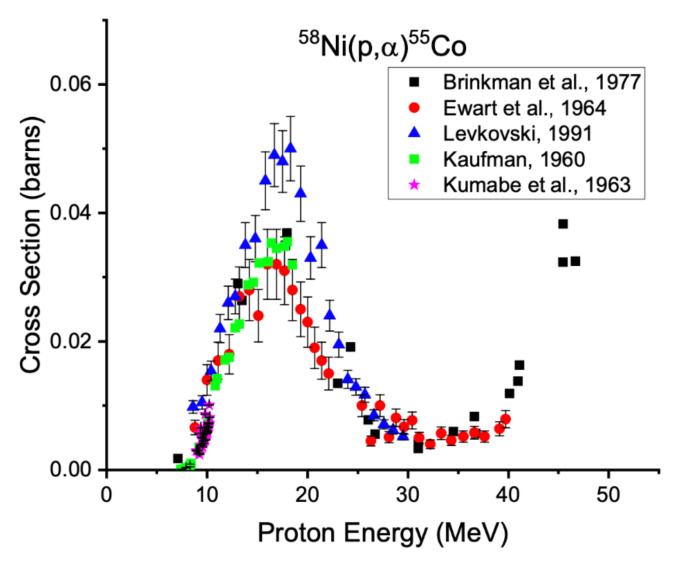
Excitation functions for production of cobalt-55 from nickel-58 [24,32,71,72,73].

**Figure 10:**
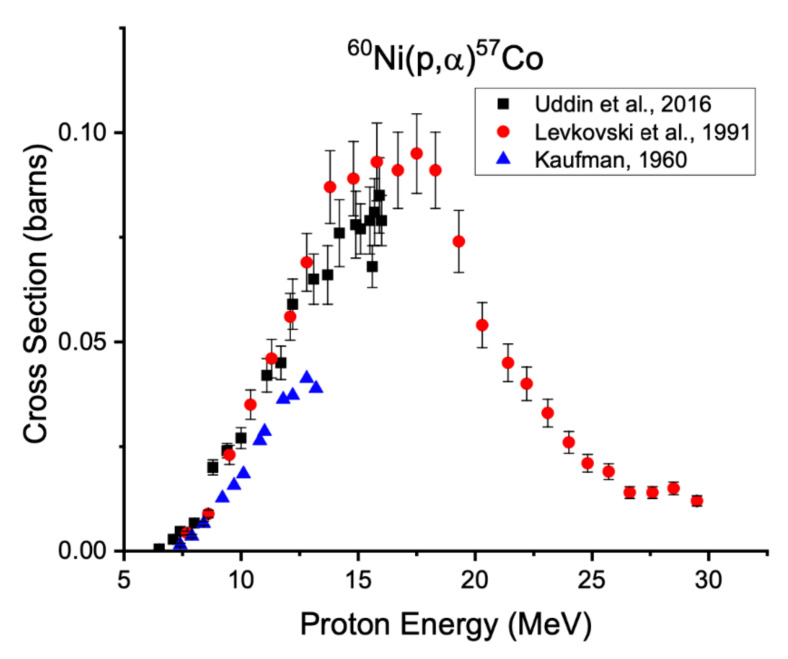
Excitation functions for production of cobalt-57 from nickel-60 [24,32,68].

**Figure 11:**
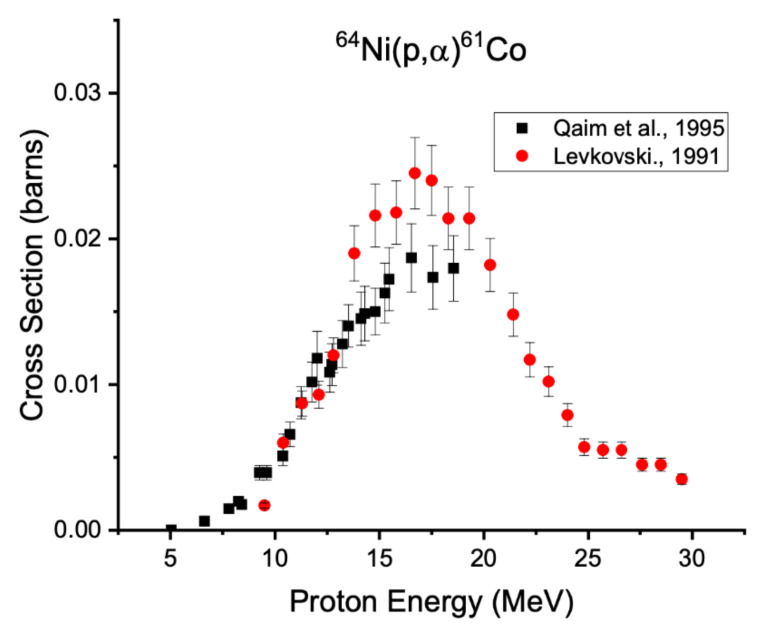
Excitation functions for production of cobalt-61 from nickel-64 [32,74].

Just as with iron targets, isotopically enriched nickel targets are required to reduce the production of radioisotopic impurities. Using enriched material prevents the production of long-lived cobalt isotopes via the (p,α) reactions on nickel-58 (68.1%), nickel-60 (26.2%), and nickel-64 (0.93%), shown in Figure 9, Figure 10 and Figure 11 below.

### 2.4. Cobalt-58m: Mn targets

**Figure 12:**
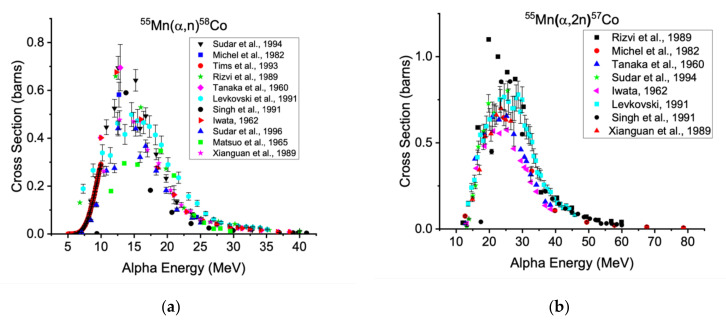
Excitation functions for the production of cobalt-58 from (α,n) reaction (**a**) and cobalt-57 (**b**) on manganese-55 [29,30,32,35,78,79,80,81,82,83,84].

Though the proton- and deuteron-induced reactions are most accessible to the majority of modern cyclotrons, there are alternative routes utilizing helium-4 ions, neutrons, or helium-3 nucleus with comparable cross sections [75,76,77]. Alpha beams afford a particularly attractive reaction route using monoisotopic manganese targets via ^55^Mn(α,n)^58m^Co. The measured excitation functions are shown below in Figure 12a.

If higher-energy alpha beams are used, cobalt-57 can be formed from ^55^Mn(α,2n), as shown in Figure 12b. To decrease the likelihood of producing this contaminant, lower-energy beams should be employed to capture the greatest cross section for the ^55^Mn(α,n)^58^Co reaction while minimizing the ^55^Mn(α,2n)^57^Co reaction.

In addition to charged particle-induced reactions, several alternative and less pragmatic routes to cobalt-58m can be contemplated, e.g., quasi-monoenergetic neutron or triton irradiations. Quasi-monoenergetic neutrons can be produced via ^2^H(d,n)^3^He reaction on hydrogen-2 gas targets. The neutrons bombard nickel or cobalt induce the ^58^Ni(n,p)^58m^Co and ^59^Co(n,2n)^58m^Co reactions [29,75]. Tritons can be generated via the ^6^Li(n,α)^3^H reaction within a nuclear reactor with an energy of 2.736 MeV. These ions go on to bombard [^56^Fe]Fe_2_O_3_ targets to produce cobalt-58m,g via the ^56^Fe(t,n)^58m^Co reaction route [76,85]. These alternative reactions currently rely on rare accelerator infrastructure or on significantly increased scale in terms of beam intensities.

**Figure 13:**
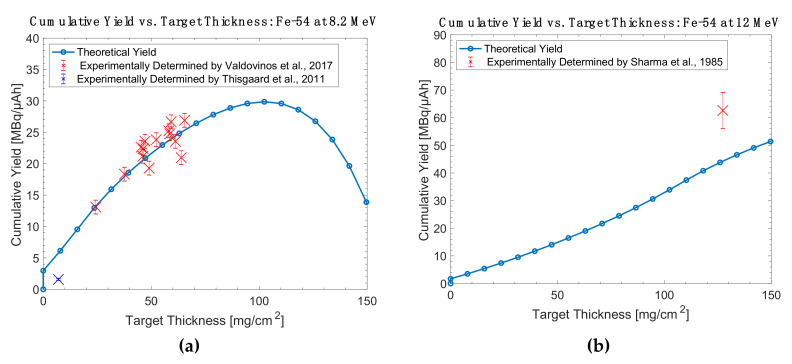
Thick target yield comparison between theoretical data and experimentally collected data for iron-54 targets at (**a**) 8.2 MeV and (**b**) 12 MeV [26,86,87].

**Figure 14:**
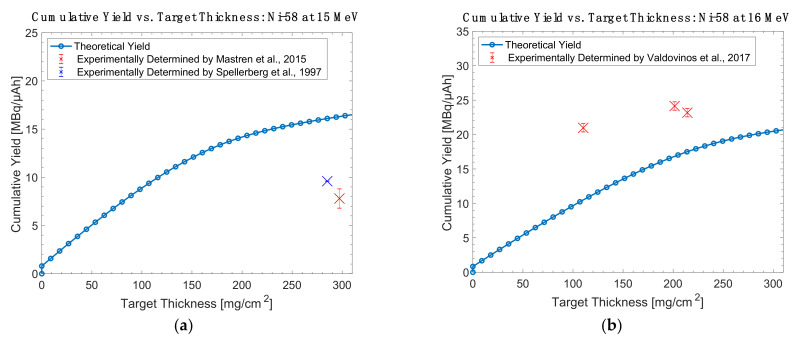
Thick target yield comparison between theoretical data and experimentally collected data for nickel-58 targets at (**a**) 15 MeV and (**b**) 16 MeV [14,23,26].

**Figure 15:**
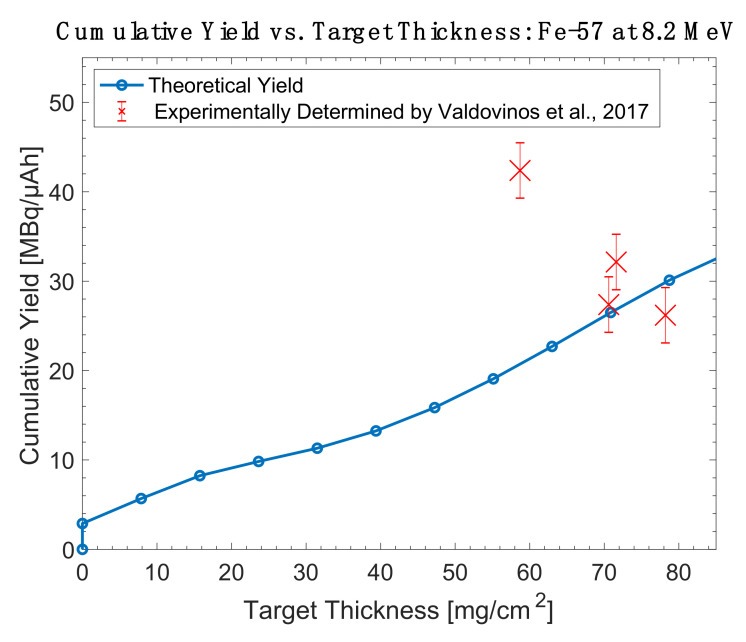
Thick target yield comparison between theoretical data and experimentally collected data for iron-57 targets at 8.2 MeV [26].

**Figure 16:**
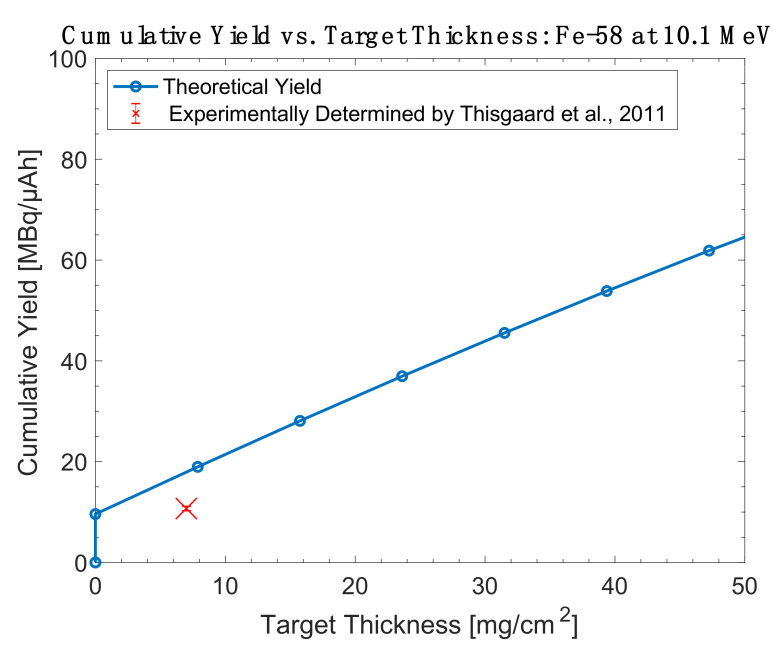
Thick target yield comparison between theoretical data and experimentally collected data for iron-58 targets at 10.1 MeV [87].

Overall, while the high cross section of ^55^Mn(α,n)^58m^Co makes it appealing, the ^58^Fe(p,n)^58m^Co and ^57^Fe(d,n)^58m^Co reactions are more practical for widespread use due to the accessibility of protons and deuterons in global accelerator installations. These are the only reactions whose literature descriptions include thick target yield measurements obtained in conjunction with radiochemical separations and radiolabeling efforts. A comparison of the experimentally determined thick target yields and theoretical yields are shown in Figure 13, Figure 14, Figure 15 and Figure 16.

As mentioned above, the co-production of cobalt-58g with cobalt-58m is unavoidable. Valdovinos et al. determined that through the ^57^Fe(d,n)^58g^Co production route, cobalt-58g has an experimental yield of 131.5 ± 19.7 KBq/µA-h at 8.2 MeV with integrated currents ranging from 90 to 124 µA-h [26]. This impurity poses an issue to the application of cobalt-58m due its long half-life and concomitant gamma emissions [88]. However, cobalt-58g can be useful for PET imaging of the biodistribution of radiolabeled agents. This potentially allows for determination of these agents’ biodistribution at timepoints many days after injection [89].

A summary of the discussed pragmatic production methods is shown in Table 2 below, highlighting the isotope produced, material needed with respective natural abundance, the reaction that must occur, and the possible radio-contaminants that may be co-produced.

Using the table above, it can be suggested that for the production of cobalt-55, iron-54 targets should be utilized based on the lack of potential long-lived radio-contaminants co-produced, and an increased yield at lower thickness targets when compared to yields produced by nickel-58.

To determine the best route for producing Co-58m, one has to consider accelerator capabilities, target material expenses and also potential co-produced contaminants. From Table 2, it would appear that producing Co-58m from Mn-51 would be the most economically feasible due to the 100% abundance of Mn-51. However, many biomedical accelerators do not have the ability to produce an alpha particle beam. Therefore, iron-57 targets are suggested to produce cobalt-58m via the d,n reaction due to the higher cross section compared to the other two methods discussed using proton irradiation. Figure 6 does not show the yield for an enriched target; the plotted value should be scaled to account for the natural abundance of iron-57.

## 3. Target Preparation

Targets of nickel and iron are relatively easy to fabricate; most literature reports describe electrochemical preparations because of the thermal and electrical conductivity of the resulting metal deposits, and presumably due to their compatibility with efforts to reuse costly separated materials.

Nickel powder, natural or enriched, can be plated onto a silver or gold backings via electrodeposition with final deposited nickel masses between 45 and 200 mg [14,26]. The procedure follows closely to the methods for plating nickel-64 for the production of copper-64 [90,91]. In a generally representative process, nickel-58 powder is dissolved in 6 M HNO_3_, dried and reconstituted in 2.3 mL of 2.4 M H_2_SO_4_. The plating solution is adjusted pH 9 using 28% NH_4_OH and 270–300 mg of (NH_4_)_2_SO_4_ and transferred to a circular electroplating cell that consists of a Teflon base that is connected to the gold coin, allowing for a 5 mm plating diameter [26]. With graphite or platinum anodes, a DC voltage of 2.5–3.6 V is applied, producing currents ranging from 8 to 116 mA. Masses of 45–55 mg are deposited in ~12 h and larger masses (100–200 mg) take 2–3 days to quantitatively electroplate onto the cathode [14,26] leaving a clear and colorless electrolyte behind.

Iron electroplating methods are largely adapted from Vosburgh et al. [89,92]. Metallic iron-5x powder is dissolved in 5 mL 6M HCl. To the dissolved iron solution, 100 µL of 30% H_2_O_2_ is added to promote the oxidation of iron 2+ ions in solution to 3+ ions. This solution is then dried to near completion, leaving <1 mL of solution in the dry down vessel. The residual solution is then diluted with 15 mL of saturated ammonium oxalate with a concentration of ~44 mg/mL H_2_O. From here, Valdovinos adjusts the pH of the solution between 2 and 3 with either 1 M NaOH or 1 M HCl [26]. However, Graves et al. uses an additional step of adding <100 mg of L-ascorbic acid to the saturated ammonium oxalate target solution to promote the reduction of iron 3+ ions to iron 2+ ions [93]. This solution is then adjusted to ~pH 3 before transferring to electroplating cell.

Thisgaard et al. explains a different method for electroplating iron powder, adapted from Zaman and Qaim [28]. In this method, iron powder is dissolved in 1 mL of H_2_SO_4_ under nitrogen-atmosphere. once dissolved, the solution is diluted with distilled water to 5 mL in order to have a pH of 1.5. This solution is then used for the plating process on a silver backing, where the current density is reported as 28–35 mA/cm^2^ for 1.5 h with a fixed voltage of 7.3 V. [87].

Another form of target fabrication is to press elemental enriched iron into 5 mm diameter pellets. Enriched iron material can come in the form of Fe_2_O_3_. If made from the oxide form, the target will have poor thermal conductivity. Therefore, the enriched Fe_2_O_3_ is reduced to elemental form by heating the powder at 800 °C for 4 h in a hydrogen tube furnace [86].

## 4. Radiochemical Isolation and Preparation

### 4.1. Anion-Exchange Chromatography

Because both cobalt-55 and cobalt-58m require isolation from iron or nickel target materials, many of the radiochemical isolation processes reported can be applied to multiple production schemes. Several anion-exchange resins such as Dowex-2, Amberlite IRA-400, and ANEX-L have been historically used for the separation of Co from various transition metals in HCl, HNO_3_, and HBr solutions [94].

Previously reported separation methods to remove nickel from cobalt used the AG 1 × 8 anion-exchange resin [14,24]. These methods included the use of multiple grams of resin packed into a glass column and equilibrated with 9 M HCl. The dissolved target solution in 9M HCl was loaded onto the column where the nickel elutes from the column in the eluate and 9M HCl wash in 10–40 mL. The cobalt-5x, which remains on the column during the large 9M HCl wash, is eluted from the column in 10 mL of 0.1 M HCl. The eluted cobalt fraction can then be evaporated to dryness and reconstituted in various matrices for further analysis and/or radiolabeling experiments.

Anion-exchange chromatography also isolates cobalt-5x from iron target material [94]. After dissolving the target in acid, the iron is oxidized into the 3+ state with hydrogen peroxide. Instead of loading the cobalt-5x on to the anion-exchange resin and washing away the target material, as was performed in the nickel/cobalt anion-exchange separation, cobalt and iron are retained on the column when loaded. The cobalt can then be eluted form the anion-exchange column in 4 M HCl. The iron target will remain on the column during the 4 M HCl wash, but can be recovered in low concentrations of HCl. The HCl eluant containing cobalt-5x can also be evaporated and the cobalt-5x reconstituted in alternative matrices without incurring significant losses [26,28,86,87].

Separation of cobalt-5x from manganese is similar to the isolation of cobalt-5x from nickel targets, but can use even less concentrated acidic media. As summarized by Bate and Leddicotte in “The Radiochemistry of Cobalt”, manganese in a dissolved target solution passes through a AG1 × 8 anion-exchange resin in HCl concentrations > 4.5 HCl. Once the target material is removed, the column can be washed with <4.5 M HCl to elute cobalt-5x [94].

### 4.2. Extraction Chromatography

As stated by Valdovinos et al., it is preferrable to use extraction chromatography instead of traditional ion exchange resins to increase separation yield and purity level [26]. This realization allowed for the implementation of N,N,N′,N′-tetrakis-2-ethylhexyldiglycolamide (DGA Branched extraction resin). The most recent published separation methods from both nickel and iron targets of varying enrichments use branched DGA to chemically isolate radioactive cobalt.

**Figure 17:**
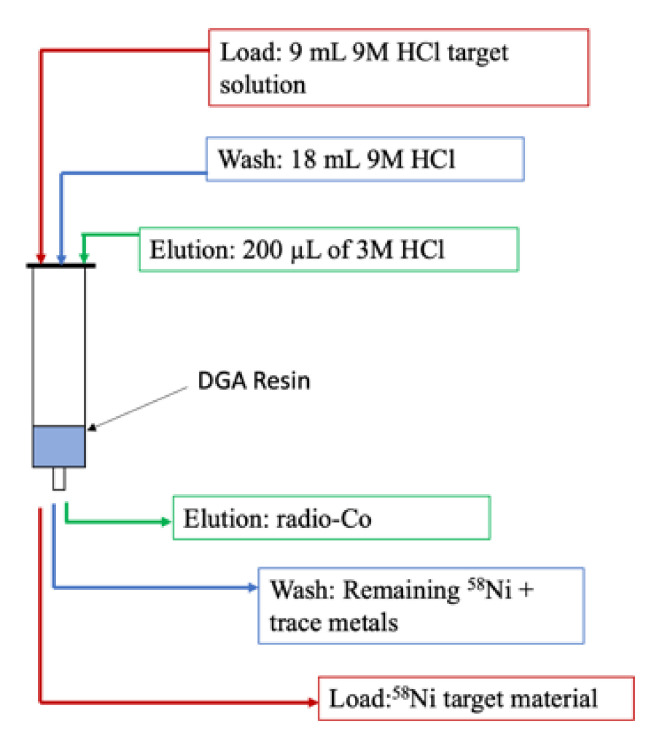
Flow diagram of the one-column method for the separation of cobalt-5x from nickel.

The newer separation method for separating nickel from cobalt is straightforward, using one column containing branched DGA resin, as shown in Figure 17.

**Figure 18:**
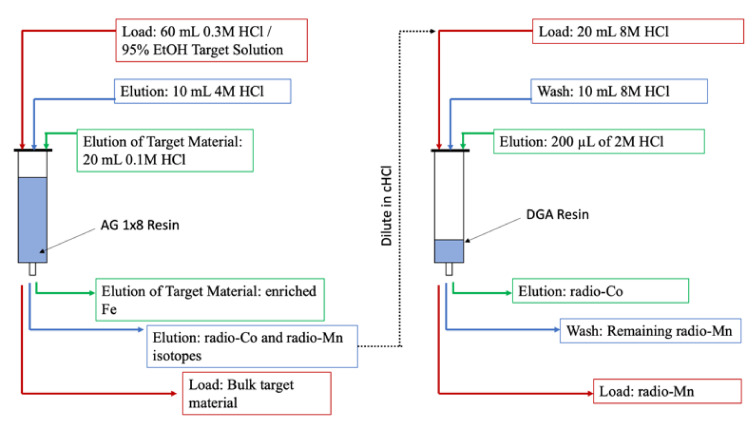
Flow diagram of the two-column method for the separation of cobalt-5x from iron.

The separation of cobalt-5x from iron-5x uses a two column technique, as shown in Figure 18. An AG1X8 anion-exchange resin packed column is used for the bulk separation of iron and cobalt, then a DGA column serves as a “clean up” to further improve the purification of cobalt-5x [26].

While there has not been a publication specifically on the separation of manganese target material from cobalt-55,58m, the work performed by Pourmand et al. suggests branched DGA can also be used for radio-chemical isolation using 4–6 M HCl, where in this range manganese 2+ has minimal affinity for the resin, and cobalt 2+ has a greater affinity [95].

### 4.3. Separation by Organic and Inorganic Absorbents

While not widely used for radiochemistry, early studies showed that cobalt can be separated from iron 3+ and nickel 2+ with the use of inorganic and organic absorbents [94]. Many of the inorganic separations were using alumina, a polar column chromatography absorbent [96]. The organic separations from iron 2+ and nickel 2+ rely on columns of 8-hydroxyquinoline, naphthaquinoline, and cupferron mixed with potato starch or dimethylglyoxime compound columns, as reported by Bate and Leddicotte, [94].

## 5. Applications of Cobalt-55,58m

### 5.1. Free Cobalt-55

Free cobalt in the form of cobalt 2+ has been found to behave in the body similarly to calcium 2+ [97]. This has been found to be especially useful in tissues damaged by ischemia as these tissues contain high levels of intracellular calcium [98]. [^55^Co]CoCl_2_ PET has been used to image areas of the brain affected by ischemic stroke, showing that [^55^Co]CoCl_2_ uptake is inversely correlated to relative cerebral blood flow (rCBF) [99]. Additionally, uptake in ischemic regions of the brain occurs regardless of whether the blood brain barrier remains intact [100].

### 5.2. Somatostatin Receptor Imaging

The somatostatin receptors (SSTRs) are a family of 5 G protein-coupled receptors that contain somatostatin-binding extracellular domains [101]. Somatostatin (SST) is a 14 amino acid peptide involved in neurotransmission and cell proliferation [101]. While most normal cell types express somatostatin receptors, several tumor types overexpress the somatostatin receptor type 2 (SSTR2) [102]. Variations of somatostatin have been developed to bind to the SSTRs for the detection and treatment of cancers. These analogs deviate from the base structure of SST in ways that increase binding specificity and plasma stability [103].

The first agent approved for PET imaging of SSTR2 was [^68^Ga]Ga-DOTATATE (Netspot) in 2016 [104]. Since its approval, there has been interest in developing a PET agent for somatostatin receptor imaging with a longer half-life than gallium-68 to simplify patient logistics and to image longer time courses post injection with improved clearance from blood and non-target tissues.

In a study published in 2008, Heppeler et al. investigated the effect of changing the metal ion on the affinity of DOTATOC for SSTR analogs and compared their results to those published by Reubi et al. [105,106]. They found that Co-DOTATOC has higher affinity than DOTATOC labeled with gallium and yttrium as well as octreotide [106]. Biodistribution studies of [^57^Co]Co-DOTATOC in mice with AR42J tumors revealed high (25% ID/g) tumor uptake at 4 h post injection (PI), even higher than the kidneys which are a clearance route for peptides. However, the tumor to kidney ratio did not increase at the 24 h timepoint. Crystallography with Ga- and Co-DOTA revealed similar pseudooctahedral geometry but differing charge states which may explain their differing binding affinities [106].

This work led to a proof-of-concept study radiolabeling DOTATOC with cobalt-55,56,58m [14]. In this study, the authors produced Co isotopes using the following reactions: ^54^Fe(d,n)^55^Co, ^nat^Fe(p,x)^56^Co, and ^58^Fe(p,n)^58m^Co. They measured molar activities of >4 MBq/nmol for [^58m^Co]Co-DOTATOC and >0.21 MBq/nmol for [^55^Co]Co-DOTATOC, but [^56^Co]Co-DOTATOC wasnwas not examined [16].

Thisgaard et al. followed this work with a comparative in vivo evaluation of ^57^Co-labeled DOTATOC, DOTANOC, and DOTATATE with [^111^In]In-DOTATATE [107]. In a head-to-head comparison in AR42J cells, the three ^57^Co-labeled somatostatin analogues were found to have between 49 and 123% higher cellular uptake as compared to [^111^In]In-DOTATATE. All agents showed similar levels of nuclear translocation, with less than 10% of nuclear translocation observed up to 3 h post addition. The cell-killing ability of the Auger emitter [^58m^Co]Co-DOTATATE was also compared to the beta emitter [^177^Lu]Lu-DOTATATE and the Auger emitter [^111^In]In-DOTATATE. At low concentrations, the [^58m^Co]Co-DOTATATE was more effective in causing DNA damage than ^111^In-labeled or ^177^Lu-labeled, while at higher concentrations the [^177^Lu]Lu-DOTATATE was most effective. When the cell-killing assay was normalized to cumulative decays, the [^58m^Co]Co-DOTATATE was consistently most effective at all concentrations tested. The authors attributed the limited degree of cell killing demonstrated by lutetium-177 to the cells being grown in monolayer, which restricts the ability of the emitted electrons to cross into adjacent cells to do damage. The superiority of ^58m^Co-over ^111^In-labeled DOTATATE may also be attributed to the higher binding affinity of the cobalt-labeled DOTATATE for SSTR2. Similarly to the 2008 study, the authors performed a biodistribution study with [^57^Co]Co-DOTATATE and found high tumor uptake 1–4 h PI. By 4 h, most uptake in organs with SSTR2 expression such as the stomach and pancreas had greatly decreased [106].

**Figure 19:**
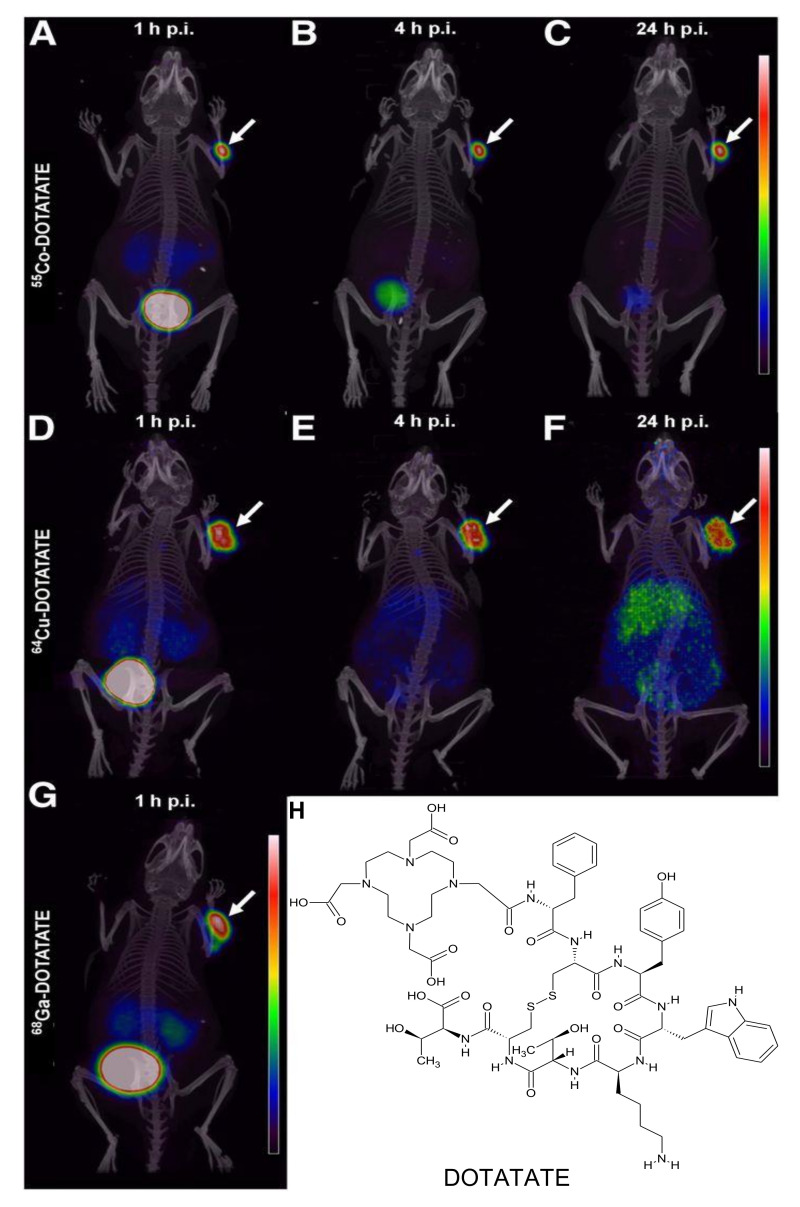
PET/CT scans comparing [^55^Co]Co-DOTATATE (**A**–**C**), [^64^Cu]Cu-DOTATATE (**D**–**F**), and [^68^Ga]Ga-DOTATATE (**G**) in AR42J tumors. Imaging showed similar tumor uptake using all 3 agents at 1 h PI. Imaging at later timepoint images showed near-complete non-target clearance using [^55^Co]Co-DOTATATE while [^64^Cu]Cu-DOTATATE showed non-specific liver uptake [15].

In a paper published by Andersen et al. in 2020 [15], [^55^Co]Co-DOTATATE was compared with [^64^Cu]Cu-DOTATATE and [^68^Ga]Ga-DOTATATE to evaluate tumor uptake and image quality. Tumor-bearing mice were imaged 1, 4, and 24 h post injection of the ^55^Co- or ^64^Cu-labeled DOTATATE, while [^68^Ga]Ga-DOTATATE imaging was only performed at 1 h post injection (PI) (see Figure 19 below). Authors found that at 1 h PI both the ^55^Co- and ^64^Cu-labeled agents showed a nonsignificant trend towards higher uptake than [^68^Ga]Ga-DOTATATE. Tumor to organ ratios increased for [^55^Co]Co-DOTATATE at the 4 h and 24 h PI imaging times, with ratios of tumor to liver, kidney, and muscle higher than [^64^Cu]Cu-DOTATATE. PET imaging showed that for all 3 agents, blood activity had mostly cleared by 1 h PI. For the [^55^Co]Co-DOTATATE, kidneys and liver did not appear on the images at 4 and 24 h PI, while liver uptake was apparent in the ^64^Cu-labeled version at 24 h PI. The authors also used the mouse biodistribution to estimate human dosimetry. They found that most organs would receive relatively low doses from [^55^Co]Co-DOTATATE (<0.2 × 10^−2^ mSv/MBq), while the lungs (6.5 × 10^−2^ mSv/MBq) and stomach (2.1 × 10^−2^ mSv/MBq) would receive higher doses.

In summary, it appears that cobalt-labeled somatostatin receptor-targeted agents have the highest affinity ever observed for SSTR2. DOTATATE labeled with cobalt-55 shows improved imaging characteristics as compared to DOTATATE labeled with copper-64 as nontarget uptake in the liver is reduced. As a therapeutic, [^58m^Co]Co-DOTATATE has shown promise as an effective auger emitter as it is has high affinity for SSTR2 and is internalized to a high degree. Using cobalt-55- and cobalt-58m-labeled DOTATATE together as a theranostic pair is an enticing prospect.

### 5.3. Gastrin-Releasing Peptide Receptor

The cell surface protein gastrin-releasing peptide receptor (GRPR) has become a popular imaging target in recent years. GRPR is overexpressed by a large number of cancers including prostate, pancreatic, colon, and non-small cell lung cancers [108]. Agonist peptides such as bombesin bind to GRPR, trigger downstream signaling and are subsequently internalized into the cell. They have higher binding affinity than antagonists and are taken up in organs with endogenous GRPR expression such as the pancreas, stomach, intestines and adrenal glands [109]. The lower binding affinity of GRPR antagonists, however, may be advantageous for imaging studies as it leads to lower uptake in normal tissues [110].

The first cobalt-55 imaging of GRPR was reported in 2016 by Dam et al. [111] and compared ^55^Co-, ^57^Co-, and ^68^Ga-labeled GRPR agonist NOTA-AMBA (NOTA-Gly-4-ABz-Gln-Trp-Ala-Gly-His-Leu-Met-NH_2_) in a PC3 prostate cancer. ^57^Co- and ^68^Ga-labeled NOTA-AMBA were found to have similar receptor affinities, serum stabilities, and pharmacokinetics at 1 h and 4 h PI [^55^Co]Co-NOTA-AMBA enabled PET imaging at a 24 h timepoint (and beyond) which is not possible with [^68^Ga]Ga-NOTA-AMBA and revealed encouraging clearance of the tracer and stable late uptake of [^5x^Co]Co-NOTA-AMBA in the tumor [111].

**Figure 20:**
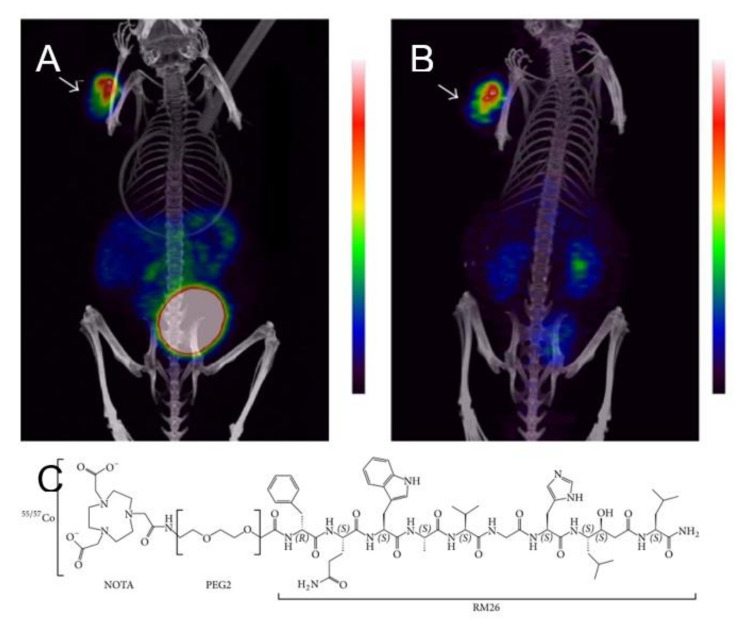
PET/CT images of [^55^Co]Co-NOTA-PEG_2_-RM26 (**C**) at 3 h PI (**A**) and 24 h PI (**B**) show the reduction in background signal between early and late timepoint imaging. [13].

In 2017, Mitran et al. reported radiolabeling GRPR antagonist RM26 (NOTA-PEG_2_-[D-Phe, Sta, Leu] bombesin [6,7,8,9,10,11,12,13,14]) with cobalt-55 and cobalt-57 [13]. Cell binding and internalization were investigated in PC3 cells and a mouse PC3 xenograft tumor model. They observed 12% cell internalization at 24 h which is similar to what has been reported for [^111^In]In-NOTA-PEG2-RM26 [112]. Biodistribution studies in PC3 tumor-bearing mice showed that at 3 h PI the tumor uptake was greater than uptake in any other organ including the kidneys. PET imaging studies using [^55^Co]Co-NOTA-PEG2-RM26 showed clear tumor uptake at 3 h PI and by 24 h PI the tumor showed high uptake (Figure 20).

The IC50 for Co-NOTA-PEG2-RM26 (5.54 ± 0.4 nM) [10] is approximately 10-fold higher than Co-NOTA-AMBA (0.46 ± 0.20 nM) [13,111]. In vivo comparisons between [^57^Co]Co-NOTA-AMBA and [^57^Co]Co-NOTA-PEG2-RM26 are complicated as the early imaging times are not matched between studies. However, comparison at the 24 h timepoint shows greater uptake of [^57^Co]Co-NOTA-AMBA than [^57^Co]Co-NOTA-PEG2-RM26 in the tumor as well as many other organs. Even though the tumor uptake was less, the tumor to organ ratios for [^57^Co]Co-NOTA-PEG2-RM26 were sometimes 100-fold greater than what was observed with [^57^Co]Co-NOTA-AMBA. This was most notable in tissues with high GRPR expression such as the colon, pancreas, and stomach. The 24 h PI PET images provided, with both papers similarly showing lower non-target organ accumulation with the [^55^Co]Co-NOTA-PEG2-RM26 than with [^55^Co]Co-NOTA-AMBA.

Recently, Mitran et al. investigated the impact chelator modification had on PEG_2_-RM26 cell binding, internalization, and biodistribution in PC-3 prostate cancer [113]. PEG_2_-RM26 was bound to NOTA, DOTA, NODAGA, or DOTAGA and radiolabeled with cobalt-55 and cobalt-57. The NOTA- and DOTA-labeled peptides showed the highest uptake in PC-3 cells in vitro and DOTA-labeled peptide had the highest tumor uptake in vivo by 24 h PI. In PET imaging studies, NOTA and DOTA PEG2-RM26 showed near-complete clearance of the radiotracer from the kidneys and GI tract by 24 h, whereas NODAGA and DOTAGA had residual GI and kidney uptake respectively.

In summary, cobalt-labeled GRPR targeting agonist and antagonist peptides successfully imaged PC3 prostate cancers. Therapy using a GRPR agonist peptide radiolabeled with cobalt-58m may be possible but would likely be limited by high uptake in non-target organs. Therapy with an antagonist remains to be determined but may suffer from low cellular internalization.

### 5.4. PSMA

More recently, bombesin analogues have been supplanted by prostate-specific membrane antigen (PSMA)-targeting agents in prostate cancer imaging, culminating in the NDA approval for [^68^Ga]Ga-PSMA-11 [114,115,116]. In 2017, Dam et al. compared ^68^Ga and ^55/57^Co-labeled PSMA-617 with in vitro-specific binding and internalization assays in LNCaP and PC3-PIP cells [12]. Observed K_D_ values for both agents were similar to [^111^In]In-PSMA-617 [117]. In LNCaP cells incubated with [^57^Co]CoPSMA-617, >60% of the cell associated radioactivity was internalized after 30 min of incubation, which was higher than ^68^Ga-, ^177^Lu-, or ^44^Sc-labeled PSMA-617 [117,118]. PET imaging with [^55^Co]Co-PSMA-617 shows selective tumor uptake by 20 min P.I. and was sustained at 24 h PI. Additionally, by 24 h, tumor uptake was found to be higher than any other organ, including the kidneys [12].

Due to the improved internalization profile and very high tumor uptake, it appears that [^58m^Co]Co-PSMA-617 would be a prime agent to be used for therapy. This is supported by its relatively rapid clearance in nontarget organs, especially the kidney.

### 5.5. Folate Receptor

Folate receptors (FR) are expressed on the surface of cells and reduce extracellular folic acid derivatives, delivering them across the cell membrane [119]. Their overexpression by many cancers makes them interesting pharmacological targets [119,120]. Radford et al. ^55^Co-labeled two folate receptor-targeting constructs, cm10 (DOTA tagged) and rf42 (NODAGA tagged) [121]. In vitro and in vivo up uptake waweres evaluated in the KB cervical cancer cell line which expresses high levels of FR [122]. The two agents had similar uptake and biodistributions, achieving >15% ID/g in xenografted tumors and underwent renal clearance [121]. Compared to a previous study using ^64^Cu- and ^68^Ga-labeled rf42, tumor to blood and tumor to kidney ratios were similar for all three agents [123]. However, tumor to liver ratios for the ^55^Co- and ^68^Ga-labeled tracers were higher than for the ^64^Cu-labeled version. Free copper is known to exhibit preferential liver uptake due to high levels of transcuprein [124].

Some of the challenges facing a therapeutic target to folate receptors include the lack of specificity towards folate receptor isoform α versus isoform β. While FRα is primarily expressed by epithelial cancers, it has high expression in the kidneys [125]. FRβ is expressed in adult tissues primarily in immune cells such as macrophages, neutrophils and monocytes [120]. Delivery of a folate receptor-targeted therapy to the tumor while sparing the kidneys has been demonstrated to be a difficult challenge [126]. The constructs cm10 and rf42 each contain an albumin-binding domain which contributes to lowering kidney uptake, which may allow them to be used therapeutically.

### 5.6. HER2/HER3

The human epidermal growth factor receptor type 2 (HER2) is a cell surface protein involved in cell growth, replication, angiogenesis and differentiation. It is overexpressed by many breast cancers, and expression status is used to determine eligibility for some therapies [127]. Detection of HER2 receptor status can be assessed with imaging, and [^89^Zr]Zr-trastuzumab has been evaluated in several clinical trials. However, when using a fully intact antibody such as trastuzumab, blood clearance is delayed and imaging is typically performed 4–5 days after injection of the radiotracer [128]. Additionally, use of full-sized antibodies for imaging results in high liver uptake as the antibodies clear through the hepatobiliary pathway. This property may result in obscurement of liver metastases. Use of a truncated antibody, such as a minibody, diabody, or fragment antibody for imaging allows for more rapid clearance, typically via the kidneys [129]. Using these constructs allows for imaging on the same day or next day, which means that a radionuclide with a shorter half-life than zirconium-89 may be preferred.

With that in mind, an affibody for HER2 (DOTA-Z_2395_-C) was developed and ^57^Co-labeled (as a substitute for cobalt-55) [130]. In vitro studies with the ^57^Co-labeled HER2 affibody revealed that radiolabeling conditions only slightly reduced immunoreactivity of the affibody. In vitro, approximately 20% of the tracer internalized over 24 h in SKOV-3 ovarian cancer cells. In vivo, biodistribution was similar to what was observed with [^111^In]In-DOTA-Z_2395_-C [131]. Blood clearance was rapid, falling from 0.5 to 0.027 % ID/g between 1 and 24 h PI. SPECT imaging of [^57^Co]Co-DOTA-Z_2395_-C at 4 h PI in mice bearing LS174T tumors (low HER2) and SKOV-3 tumors (high HER-2) revealed blockable tumor uptake and kidney clearance [130].

Like HER2, HER3 is overexpressed by certain cancer types. Additionally, HER3 expression changes in response to therapy, increasing in tissues as they become resistant to HER2 targeted therapies [132]. As immunotherapies to HER3 are developed, it becomes important to develop tools to detect HER3 status in tumors [133]. Hence, an affibody which targets HER3 (HEHEHE-Z_HER3_) was conjugated with NOTA and radiolabeled with ^57^Co [134]. In vitro, incubation of [^57^Co]Co-NOTA-Z_HER3_ with cells expressing low HER3 (DU145) and high HER3 (LS174T) showed better total binding to LS174T (~15% vs. 4% for DU145). Internalization was observed to be higher with the DU145 cells, with approximately 50% of activity internalized by DU145 at 24 h, and less than 10% internalization seen with LS174T. As expected, the high expression line LS174T showed greater tumor binding in vivo, which was effectively blocked with non-labeled Z_HER3_. Uptake was also seen in tissues which have endogenous expression of HER3, namely the liver and intestines. Clearance occurred primarily through the kidneys. By 24 h PI, imaging showed that much of the activity in the liver and intestines had cleared [134].

**Figure 21:**
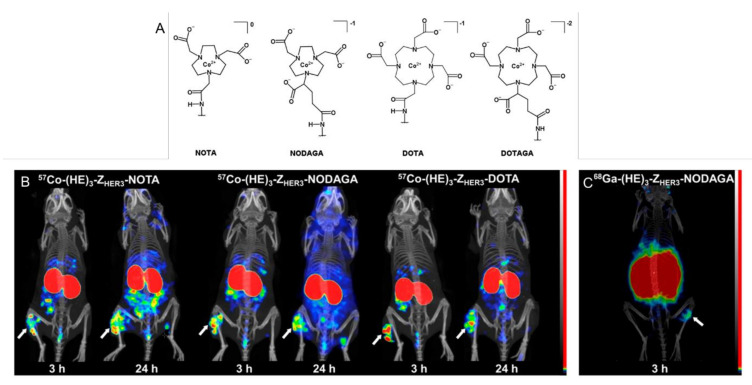
Chemical structures and charges of studied cobalt-chelate complexes (**A**). SPECT/CT images of the [^57^Co]Co-HEHEHE-Z_HER3_-X affibody with NOTA, NODAGA, or DOTA chelator (**B**). For each agent tested, the later imaging timepoint produced better tumor contrast. PET/CT of [^68^Ga]Ga-HEHEHE-Z_HER3_-NODATA was only possible to 3 h PI and showed greater kidney accumulation (**C**) [135].

Further investigation into the Z_HER3_ affibody involved modifying the chelator to determine if imaging qualities could be improved [135]. In addition to NOTA, which was used previously, DOTA-, NODAGA-, and DOTAGA-tagged Z_HER3_ were ^57^Co labeled. In vitro assays showed nearly equal, blockable binding of the affibody conjugated DOTA, NODAGA, and DOTAGA. The measured equilibrium disassociation constants, K_D_, were 0.2 nM for DOTA and DOTAGA constructs and 0.1 nM for the NOTA and NODAGA constructs. Interestingly, the internalization fraction of [^57^Co]Co-NOTA-Z_HER3_ in DU145 cells was observed to be 5% over 24 h, while their previous study showed as compared to 50% in their previous study [134]. In vivo, [^57^Co]Co-(NOTA/NODAGA/DOTA/DOTAGA)-Z_HER3_ were compared at 3 h and 24 h PI. The DOTA- and DOTAGA-conjugated affibodies showed more rapid blood clearance than those labeled with NOTA and NODAGA. [^57^Co]Co-DOTA-Z_HER3_ was compared to [^68^Ga]Ga-NODAGA-Z_HER3_ at 3 h post injection and [^57^Co]Co-DOTA-Z_HER3_ was followed to 24 h (see Figure 21 below). At 3 h, [^68^Ga]Ga-NODAGA-Z_HER3_ showed similar tumor uptake but lower blood, liver, and small intestine uptake. By 24 h, [^57^Co]Co-DOTA-Z_HER3_ showed similar tumor to organ ratios as ^68^Ga at 3 h PI [135].

The anti-HER2 [^57^Co]Co-DOTA-Z_2395_-C and anti HER3 [^57^Co]Co-Z_HER3_ affibodies showed promising tumor uptake, rapid blood clearance, and good imaging properties in the models tested. They would very likely translate well for labeling with ^55^Co for PET imaging. As a potential therapeutic, a ^58m^Co-labeled HER2/HER3 affibody may not show significant internalization needed for effective therapeutic response.

### 5.7. EGFR

The epidermal growth factor receptor (EGFR) is a transmembrane protein involved in cell growth and replication. It is upregulated in many types of cancer and is involved in resistance to chemotherapies [136]. Imaging can determine EGFR receptor status for targeted therapies and for predicting treatment resistance [136]. The EGFR-targeting affibody Z_EGFR:2377_ was conjugated with DOTA for imaging studies and ^111^In-, ^68^Ga-, or ^57^Co-labeled [137,138]. [^68^Ga]Ga- DOTA-Z_EGFR:2377_ and [^57^Co]Co-DOTA-Z_EGFR:2377_ uptake was measured in mice bearing A431 tumors. Interestingly [^57^Co]Co-DOTA-Z_EGFR:2377_ outperformed [^68^Ga]Ga-DOTA-Z_EGFR:2377_ at 3 h PI, showing higher tumor uptake and lower liver and spleen uptake. When biodistribution was performed in mice injected with [^57^Co]Co-DOTA-Z_EGFR:2377_ at 24 h PI, a reduction in blood activity was observed and was accompanied by only minor reduction in activity in other organs. Similarly, imaging showed much less liver uptake using the ^57^Co-labeled agent than the ^68^Ga-labeled agent. This radionuclide-dependent uptake led the authors to speculate that liver binding may be due to more than EGFR targeting in the liver. Because their blocking dose only partly blocked liver binding, they theorize that the neutral charge on the Ga-DOTA complex gives it higher affinity for scavenger receptors in the liver. The reduction in liver binding may leave more affibody available for tumor binding, explaining the higher tumor binding observed with [^57^Co]Co-DOTA-Z_EGFR:2377_.

Attempts to produce a therapeutic compound labeled with ^58m^Co would likely have to contend with poor intracellular internalization and high kidney uptake, similar to the challenges faced in the development of PSMA-targeted ligands. Additionally, EGFR targeting would cause a significant radioactive dose to the liver.

### 5.8. VEGF

Vascular endothelial growth factor (VEGF) helps promote vascular development in tissues. overexpression of VEGF is associated with a poor prognosis in some types of cancer [139,140]. A VEGF-targeting peptide L19K-FDNB was conjugated to NO2A or DO3A and labeled with cobalt-55 for PET imaging [14]. Uptake of the radiotracers was compared with the biodistribution of free cobalt. In tumor-bearing mice, free cobalt distributed primarily to the liver, kidneys, and heart while the ^55^Co-peptides were observed to clear through the kidneys. Unexpectedly, uptake in the tumors was highest for free cobalt, though it is unclear whether this is due to Ca^2+^ mimetic properties of Co or higher blood activity seen with free cobalt than the labeled peptides. The group had previously investigated [^64^Cu]Cu-NO2A-L19K-FDNB which showed higher tumor uptake than the cobalt-labeled peptides [141], which indicates that the affinity of this peptide for targeting VEGF was negatively affected by radiolabeling with cobalt-55.

## 6. Conclusions

A summary of molar activities obtained for the targeting paradigms discussed above is included in Table 3 below. Cobalt-55 and cobalt-58m are ideally suited as a matched-pair theranostic for use in both research and clinical environments. A wide range of production pathways are available for cobalt-55 and cobalt-58m with distinct advantages to each method. For use in clinical applications, enriched targets coupled with modern cyclotrons are likely the best path forward since this method can result in high radiochemical purities (>98%). Furthermore, the literature for chelating cobalt-58m was reviewed and results compared with clinically approved and other contemporary radiopharmaceuticals. In many of the applications reported, cobalt-55 and cobalt-58m are at least comparable to gallium-68 and lutetium-177 with some studies also reporting superior contrast with cobalt-55 over gallium-68 and higher cell-killing efficiencies with cobalt-58m as compared to lutetium-177. However, despite the favorable results, there have not been any in vivo human trials using cobalt-55 and cobalt-58m in targeted radionuclide therapy and thus further studies are necessary.

## Figures and Tables

**Table 1:** Nuclear reactions, thresholds, and Q-values for the production of Co radioisotopes from ^58^Fe targets.

Reaction	Threshold [MeV]	Q-Value [MeV]
^58^Fe(p,n)^58^Co	3.14 ± 0.04	−3.09 ± 0.03
^58^Fe(p,2n)^57^Co	11.87 ± 0.06	−11.66 ± 0.06
^58^Fe(p,3n)^56^Co	23.44 ± 0.14	−23.04 ± 0.14

**Table 2:** Summary of alternative reactions which reduce the radionuclidic purity of ^55^Co and ^58m^Co.

Desired Isotope	Target Material	Abundance	Reaction	Likely Contaminating Reactions
Cobalt-55	Nickel-58	68.1%	p,α	^58^Ni(p,2p)^57^Co, ^58^Ni(p,2n)^57^Cu → ^57^Ni → ^57^Co, ^60^Ni(p,α)^57^Co
Cobalt-55	Iron-54	5.8%	d,n	^54^Fe(d,α)^52m/g^Mn
Cobalt-58m	Iron-58	0.3%	p,n	^58^Fe(p,2n)^57^Co, ^58^Fe(p,3n)^56^Co
Cobalt-58m	Iron-57	2.2%	d,n	^57^Fe(d,2n)^57^Co
Cobalt-58m	Nickel-61	3.6%	p,α	^60^Ni(p,α)^57^Co
Cobalt-58m	Manganese-55	100%	α,n	^55^Mn(α,2n)^57^Co

**Table 3:** Summary of molar activities and reaction conditions obtained for ^55/58m^Co with various targeting agents.

Targeting Agent	Molecular Target	Radionuclide	Radionuclide Source	Labeling Buffer	Labeling Condition	Molar Activity	Source
DOTATATE	SSTR2	Cobalt-55	^54^Fe(d,n)^55^Co	NaOAc pH 4.6	2 min microwave irradiation	17.5 MBq/nmol	[15]
DOTATOC	SSTR2	Cobalt-55	^54^Fe(d,n)^55^Co	NaOAc pH 4.5	80–85 °C, 30 min	0.21 MBq/nmol	[16]
DOTATOC	SSTR2	Cobalt-58m	^58^Fe(p,n)^58m^Co	NaOAc pH 4.5	80–85 °C, 30 min	4 MBq/nmol	[16]
DOTA-PSMA-617	PSMA	Cobalt-55	^54^Fe(d,n)^55^Co	NaOAc pH 4.4 0.4 M	2 min microwave irradiation	18.4 MBq/nmol	[12]
DO3A/NO2A-L19K-FDNB	VEGF	Cobalt-55	^58^Ni(p,α)^55^Co	Et_3_NOAc pH 6 0.1 M	37 °C, 20 min	* 11 MBq/nmol	[14]
NOTA-Peg2-RM26	GRPR	Cobalt-55	^54^Fe(d,n)^55^Co	NH_4_OAc pH 5.5 0.2 M	1 min microwave irradiation 850 W	* 16 MBq/nmol	[13]
NOTA-AMBA	GRPR	Cobalt-55	^54^Fe(d,n)^55^Co	NaOAc pH 4.6	1 min microwave irradiation 850 W	34 MBq/nmol	[12]
cm10/ rf42	Folate Receptor	Cobalt-55	^58^Ni(p,α)^55^Co	NH_4_OAc pH 6 0.5 M	50 °C, 30 min	1.3 MBq/nmol	[121]
DOTA-Z_EGFR:2377_	EGFR	Cobalt-55	^54^Fe(d,n)^55^Co	NH_4_OAc pH 6	60 °C, 30 min	7 MBq/nmol	[138]
DOTATOC	SSTR2	Cobalt-57	N/A	NaOAc pH 5 0.4M	95 °C, 30 min	* 0.22 MBq/nmol	[106]
DOTA-Z_2395_-C	Her2	Cobalt-57	N/A	NH_4_OAc pH 5.5 0.2 M	60 °C, 10 min	* 0.17 MBq/nmol	[130]
(NOTA)-Z_HER3_	HER3	Cobalt-57	N/A	NH_4_OAc pH 5.5	60 °C, 10 min	0.7 MBq/µg	[135]

* Molar activity calculated from provided methods.
